# Mapping the Binding Site of the Inhibitor Tariquidar That Stabilizes the First Transmembrane Domain of P-glycoprotein*

**DOI:** 10.1074/jbc.M115.695171

**Published:** 2015-10-26

**Authors:** Tip W. Loo, David M. Clarke

**Affiliations:** From the Departments of Medicine and Biochemistry, University of Toronto, Toronto, Ontario M5S 1A8, Canada

**Keywords:** ABC transporter, membrane enzyme, membrane protein, protein cross-linking, protein folding

## Abstract

ABC (ATP-binding cassette) transporters are clinically important because drug pumps like P-glycoprotein (P-gp, ABCB1) confer multidrug resistance and mutant ABC proteins are responsible for many protein-folding diseases such as cystic fibrosis. Identification of the tariquidar-binding site has been the subject of intensive molecular modeling studies because it is the most potent inhibitor and corrector of P-gp. Tariquidar is a unique P-gp inhibitor because it locks the pump in a conformation that blocks drug efflux but activates ATPase activity. *In silico* docking studies have identified several potential tariquidar-binding sites. Here, we show through cross-linking studies that tariquidar most likely binds to sites within the transmembrane (TM) segments located in one wing or at the interface between the two wings (12 TM segments form 2 divergent wings). We then introduced arginine residues at all positions in the 12 TM segments (223 mutants) of P-gp. The rationale was that a charged residue in the drug-binding pocket would disrupt hydrophobic interaction with tariquidar and inhibit its ability to rescue processing mutants or stimulate ATPase activity. Arginines introduced at 30 positions significantly inhibited tariquidar rescue of a processing mutant and activation of ATPase activity. The results suggest that tariquidar binds to a site within the drug-binding pocket at the interface between the TM segments of both structural wings. Tariquidar differed from other drug substrates, however, as it stabilized the first TM domain. Stabilization of the first TM domain appears to be a key mechanism for high efficiency rescue of ABC processing mutants that cause disease.

## Introduction

Human ATP-binding cassette (ABC)^2^ transport proteins mediate the ATP-dependent flux of substrates across extra- or intracellular membranes including metabolic products, lipids, sterols, ions, and drugs. Multidrug resistance in cancer, cystic fibrosis (CF), gout, intrahepatic cholestasis types 2 and 3, Tangier disease, Dubin-Johnson syndrome, hyperinsulinimic hypoglycemia of infancy, pseudoxanthoma elasticum, secretory diarrheas, gout, anemia, diabetes, and atherosclerosis are potentially lethal or debilitating conditions caused by mutations or altered activity of one of the 48 human ABC (ATP-binding cassette) proteins (1).

Considerable efforts have been made to achieve two clinical goals: 1) turn off ABC drug pumps such P-gp (ABCB1), BCRP (ABCG2), and MRP1 (ABCC1) to prevent multidrug resistance; and 2) repair folding mutants of CFTR (ABCC7; cystic fibrosis), BCRP (ABCG2; gout), ABCB4/ABCB11 (progressive familial intrahepatic cholestasis), ABCA1 (Tangier disease), ABCC2 (Dubin-Johnson syndrome), ABCC6 (pseudoxanthoma elasticum), and ABCC8 (hyperinsulinimic hypoglycemia of infancy) using small molecules called correctors or pharmacological chaperones. Large scale screening efforts have been promising but most compounds identified to date have been poor therapeutic candidates as they only caused partial inhibition of the pump or rescue of misfolded ABC proteins.

Studying P-gp interaction with tariquidar is important for understanding how to turn off drug pumps or rescue misfolded mutants. Tariquidar is different from previous inhibitors because it is not transported by P-gp but binds to the protein to activate ATPase activity (2). Although tariquidar is the most effective inhibitor identified to date, it still needs to be improved as clinical trials suggest that inhibition of transport is not complete (3). It appears that P-gp needs to be almost completely inactivated to improve therapy because it works so rapidly and with such high capacity that it can still prevent entry of substrates even when most of the pumps are turned off (4).

Tariquidar is also the most potent P-gp corrector as processing mutants could be rescued by carrying out expression in 100 nm tariquidar (2). Rescue of P-gp processing mutants with other drug substrates (5) or rescue of other ABC proteins with correctors requires at least 10-fold higher concentrations (6).

To understand P-gp/tariquidar interactions, many *in silico* docking studies have been performed to identify the drug-binding sites (7–12). In retrospect, some of the earlier docking and molecular dynamic studies done with human P-gp homology models were suboptimal because they were based on the crystal structures of ABC transporters from *Staphylococcus aureus* bacteria (Sav1866) (11), *Caenorhabditis elegans* (12), or an earlier mouse structure (13). There is low sequence homology between Sav1866 and human P-gp in the TMDs, the structure of TM10 in *C. elegans* P-gp is undefined, whereas the earlier P-gp structure from mouse was subsequently found to contain quite a few errors (14–17). *In silico* docking (8, 9) and molecular dynamics studies (9) have recently been done with homology models of human P-gp based on the corrected crystal structures of P-gp from mouse (16). For example, McCormick *et al.* (9) performed molecular dynamic simulations of human P-gp to show transport of two different substrates through the plane of the membrane. By contrast, tariquidar did not show this movement but stabilized P-gp in an outward open conformation. They also identified three potential tariquidar-binding sites. Therefore, one of our goals was to biochemically test these predictions.

We previously used alanine scanning mutagenesis to map the locations of substrate-binding sites in a membrane transport protein (SERCA1 Ca-ATPase) (18). A problem with using alanine-scanning mutagenesis to map the location of drug-binding sites in P-gp was that introduction of a small side chain in the drug-binding pocket caused little detectable effect on binding of relatively large drug substrates (19, 20). Another problem is that a small change to alter the hydrophobicity of a side chain simply changes the substrate specificity of P-gp (21).

Here, we used cross-linking protection assays and arginine mutagenesis of residues within the 12 TM segments to test for residues close to or within the tariquidar-binding site. The rationale for arginine mutagenesis was that insertion of a bulky charged side chain into a tariquidar-binding site would inhibit tariquidar rescue of processing mutants and tariquidar-stimulated ATPase activity. Our results suggest that tariquidar binds to a site within the drug-binding pocket because 30 arginines introduced into the TM segments disrupted both tariquidar rescue of processing mutants and tariquidar-stimulated ATPase activity. Unlike other drug substrates, tariquidar promoted maturation and stabilized the first transmembrane domain (TMD1). Stabilization of TMD1 may be an important mechanism in rescuing misfolded ABC proteins because a similar mechanism appears to be involved in the rescue of misprocessed CFTR protein by the corrector VX-809 (22).

## Experimental Procedures

### 

#### 

##### Construction of Mutants

Mutations were introduced into the wild-type, Cys-less, or G251V P-gp cDNAs (residues 1–1280) containing the A52-epitope or 10-histidine tags (23) by site-directed mutagenesis as described by Kunkel (24). For the arginine-scanning mutagenesis and tariquidar rescue study of TM segments 1–12, the cDNA of mutant G251V P-gp was modified to contain an arginine at positions Thr^55^-Phe^72^ (TM1), Ser^119^-Cys^137^ (TM2), Lys^189^-Val^206^ (TM3), Leu^214^-Trp^232^ (TM4), Thr^294^-Ala^311^ (TM5), Val^331^-Ala^348^ (TM6), Val^712^-Phe^732^ (TM7), Phe^759^-Phe^777^ (TM8), Lue^833^-Ser^850^ (TM9), Leu^857^-Val^874^ (TM10), Phe^938^-Gly^955^ (TM11), or Val^974^-Ser^992^ (TM12). Mutants were constructed to contain an A52 epitope tag at their C-terminal ends for use in whole cell immunoblot assays (25). The presence of the epitope tag distinguished the mutant proteins from any endogenous P-gp. P-gp contains three *N*-linked glycosylation sites that can be used to monitor maturation of human P-gp from an immature 150-kDa protein to a mature 170-kDa protein.

When an arginine mutation was identified that inhibited rescue with tariquidar, it was then introduced into the histidine-tagged wild-type P-gp cDNA. The P-gp cDNA was modified to contain a 10-histidine tag at the COOH-terminal end to facilitate purification of the expressed protein by nickel-chelate chromatography (26). Truncation mutants consisting of residues 1–379 (TMD1) or 681–1025 (TMD2) that contained the epitope for monoclonal antibody A52 at the C-terminal end were constructed as described previously (27).

##### Expression and Maturation of G251V Arginine Mutants

The G251V mutant cDNAs were transiently expressed in HEK 293 cells by a calcium phosphate precipitation approach as described previously (28). Briefly, 10 μl of 2.5 m CaCl_2_ was added to 90 μl of H_2_O containing 2 μg of DNA followed by addition of 100 μl of BES solution (50 mm BES, 280 mm NaCl, and 1.5 mm Na_2_HPO_4_, pH 6.96). After 10 min at room temperature, 4 ml of HEK 293 cells (about 100,000 cells/ml) in Dulbecco's modified Eagle's medium (DMEM) with high glucose (supplemented with nonessential amino acids, 4 mm l-glutamine, 10 IU/ml of penicillin, 10 μg/ml of streptomycin, and 10% (v/v) bovine calf serum) was added and 1.5 ml of the mixtures were added to duplicate well of 6-well culture plates. After 4 h at 37 °C, the medium was replaced with fresh medium with or without 0.5 μm tariquidar (UHN (Shanghai) Research & Development Co., Toronto, ON). About 16 h later, the cells were harvested, washed with PBS, and cell pellets were suspended in 150 μl of 2× SDS sample buffer (125 mm Tris-HCl, pH 6.8, 4% (w/v) SDS, 4% (v/v) 2-mercaptoethanol containing 25 mm EDTA). Samples were applied to 6.5% SDS-PAGE gels (minigels, 1.5 mm spacers, 15 wells). The gels were electroblotted onto a sheet of nitrocellulose and P-gp proteins were detected using A52 monoclonal antibody, horseradish peroxidase-conjugated anti-mouse secondary antibody, and enhanced chemiluminescence (Luminata Forte, Millipore Corp., Etobicoke, ON). An equivalent amount of the sample was also loaded onto 10% (v/v) SDS-PAGE gels and subjected to immunoblot analysis with a monoclonal antibody against glyceraldehyde-3-phosphate dehydrogenase (GADPH) (internal control). The signals were imaged and relative levels of immature (150 kDa) and mature (170 kDa) P-gp were determined using Chemidoc^TM^ XRS^+^ with Image Lab^TM^ software (Bio-Rad).

##### Disulfide Cross-linking Analysis

Cys-less P-gp mutants containing pairs of cross-linkable cysteines in the TM segments (A80C(TM1)/R741C(TM7), I299C(TM5)/F770C(TM8), and T333C(TM6)/L975C(TM12)), intracellular loops (ICLs) (L175C(ICL1)/N820C(ICL3), A259C(ICL2)/W803C(ICL3), and A266C(ICL2)/F1086C(NBD2)), or the NBDs (C431(NBD1)/L1176C(NBD2), L531C(NBD1)/C1074(NBD2), and P517C(NBD1)/I1050C(NBD2)) were transiently expressed in HEK 293 cells at reduced temperature (30 °C) to promote maturation. Cells expressing mutant A80C(TM1)/R741C(TM7) were washed with PBS containing 10 mm dithiothreitol before membrane preparation because the mutant cross-links spontaneously (29). Membranes were prepared and samples were incubated at 0 °C (A80C/R741C, A259C/W803C, I299C/F770C, and A266C/F1086C) or 20 °C (L175C/N820C, C431/L1176C, and L521C/C1074) for 10 min in the presence or absence of 0.5 mm copper phenanthroline (oxidant to promote disulfide bond formation) in the presence or absence of 0.25 μm (for TM segment cysteine mutants) or 1 μm tariquidar (for the ICL and NBD cysteine mutants). Mutant T333C/L975C was cross-linked in whole cells at 20 °C with 0.5 mm BMOE (bismaleimidoethane) cross-linker (Thermo Fisher Scientific, Burlington, ON) in the absence or presence of 0.25 μm tariquidar (30). Mutant P517C/I1050C was cross-linked in membranes at 0 °C with 50 μm 1,4-butanediyl bismethanethiosulfonate (BMTS) in the absence or presence of 1 μm tariquidar (30). The reactions were stopped by addition of 2× SDS sample buffer (125 mm Tris-HCl, pH 6.8, 20% (v/v) glycerol, and 4% (w/v) SDS) containing 25 mm EDTA and no reducing agent. The reaction mixtures were then subjected to SDS-PAGE (6.5% (w/v) polyacrylamide gels and 7% gels for mutant A80C/R741C) and immunoblot analysis with a rabbit polyclonal antibody against P-gp. Intramolecular disulfide cross-linking between domains can be detected because the cross-linked product migrates with a slower mobility on SDS-PAGE gels (31).

##### Purification of P-gp and Measurement of ATPase Activity

Histidine-tagged wild-type P-gps containing arginine mutations were expressed in HEK 293 cells in the presence of 10 μm cyclosporine A at 37 °C in the presence of 5 mm sodium butyrate for 24 h and then incubated at 30 °C for 24 h. Sodium butyrate is a histone deacetylase inhibitor that enhances expression of proteins in HEK 293 cells (32). Maturation of the mutants was promoted by carrying out expression at low temperature in the presence of cyclosporine A. The rescued P-gp mutants were isolated by nickel-chelate chromatography as described previously (26). Recovery of P-gp was monitored by immunoblot analysis with rabbit anti-P-gp polyclonal antibody (33). A sample of the isolated histidine-tagged P-gp was mixed with an equal volume of 10 mg/ml of sheep brain phosphatidylethanolamine (Type II-S, Sigma) that had been washed and suspended in TBS. ATPase activity was measured in the presence of various concentrations of tariquidar.

##### Endoglycosidase and Trypsin Digestion of TMD1

HEK 293 cells in 6-well plates were transfected with cDNAs of A52-tagged TMD1 and A52-tagged TMD2 and expressed for 16 h in the absence or presence of 1 μm tariquidar. The cells were harvested, washed once with PBS, and solubilized with 0.2 ml of SDS sample buffer. Samples were run on 10% SDS-PAGE gels and subjected to immunoblot analysis with monoclonal antibody A52.

In experiments involving endoglycosidase digestion, cells were co-transfected with cDNAs of A52-tagged TMD1 and untagged TMD2 and expressed in the presence of 1 μm tariquidar. The cells were harvested, washed once with PBS, and suspended in PBS. The cells were solubilized by addition of 0.1 volume of 10× denaturation buffer (5% (w/v) SDS, 100 mm EDTA, and 10% (v/v) 2-mercaptoethanol). For treatment with endoglycosidase H_f_ (New England Biolabs, Whitby, ON), 0.1 volume of 0.5 m sodium citrate, pH 5.5, was added to a sample of the solubilized cells followed by addition of endoglycosidase H to a final concentration of 20,000 units/ml. For treatment with endoglycosidase F (PNGase F; New England Biolabs, Whitby, ON), 0.1 volume of 0.5 m sodium phosphate buffer, pH 7.5, and 10% (v/v) Triton X-100 were added to sample of the solubilized cells followed by addition of PNGase F to a final concentration of 10,000 units/ml. The samples were incubated for 15 min at 37 °C and the reactions stopped by addition of 1 volume of 2× SDS buffer. The samples were run on 10% SDS-PAGE gels and subjected to immunoblot analysis.

Digestion with TPCK-trypsin was done on membranes (27) prepared from HEK 293 cells that co-expressed A52-tagged TMD1 and untagged TMD2 in the absence or presence of 1 μm tariquidar. The membranes were suspended in Tris-buffered saline, pH 7.4 (5 mg/ml of protein). Samples of the membranes were treated for 5 min at 20 °C with various concentrations of TPCK-trypsin (12,000 BAEE units/mg; Sigma). The reactions were stopped by addition of lima bean trypsin inhibitor (Worthington Biochemical Corp.) followed by 1 volume of 2× SDS buffer. The samples were run on 10% SDS-PAGE gels and subjected to immunoblot analysis.

##### Data Analysis

The signals from the immunoblots were visualized and quantified using the ChemiDoc^TM^ XRS^+^ with Image Lab^TM^ software (Bio-Rad). The results were expressed as an average of triplicate experiments ± S.D. The Student's two-tailed *t* test was used to determine statistical significance (*p* < 0.001).

## Results

### 

#### 

##### Tariquidar Inhibits Cross-linking between Cysteines Located in the TM Segments

**FIGURE 1.**
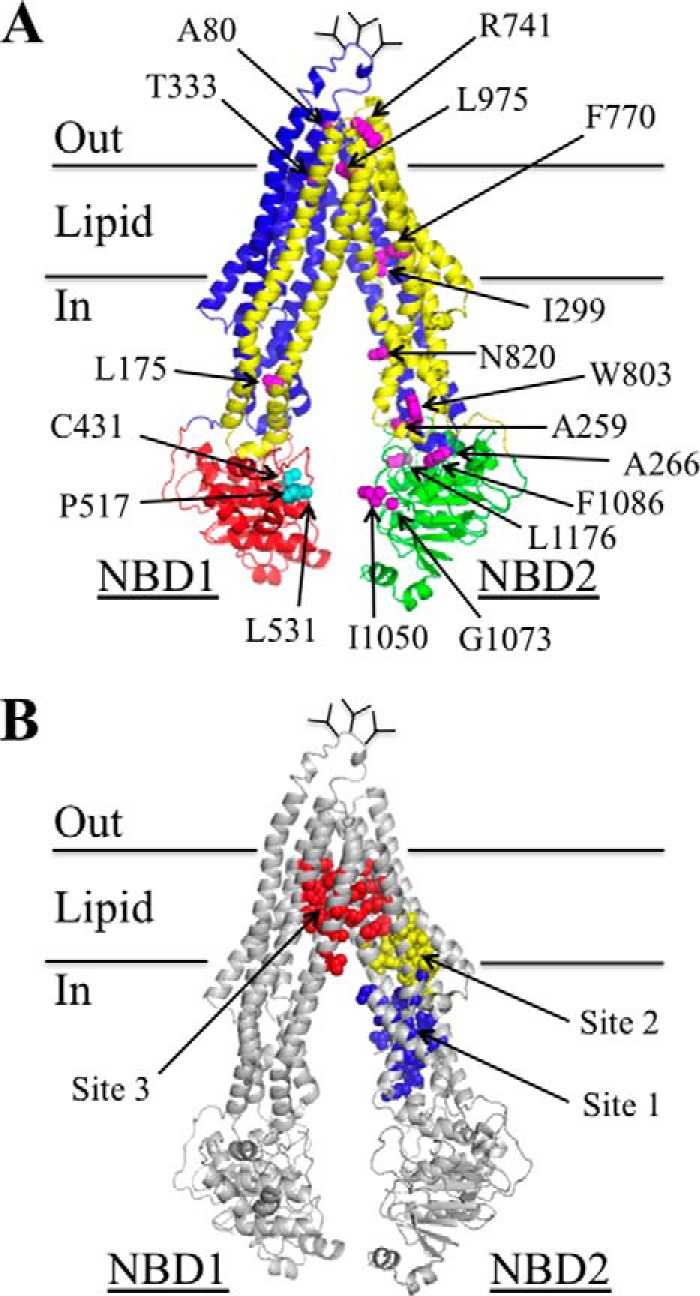
**The three predicted tariquidar binding sites.** *A,* predicted structure of human P-gp in an open conformation (NBDs apart and drug-binding pocket closed at the extracellular surface) based on the crystal structure of mouse P-gp (16). The model was viewed using the PyMol system (42). TMD1 is shown in *blue* and TMD2 is shown in *yellow*. The branched lines between TM segments 1 and 2 represent glycosylated sites. The locations where cross-linkable cysteines were introduced into the TM segments (A80C(TM1)/R741C(TM7), I299C(TM5)/F770C(TM8), and T333C(TM6/L975C(TM12)), ICL2/ICL3 (L175C(ICL1)/N820C(ICL3), A259C(ICL2)/W803C(ICL3), and A266C(ICL2/F1086C(NBD2)) or the interface between NBD1 and NBD2 (C431(NBD1)/L1176C(NBD2), L531C(NBD1)/C1074(NBD2), and P517C(NBD1)/I1050C(NBD2)) are indicated. *B,* the *colored spheres* represent the residues in predicted (9) tariquidar-binding sites 1 (*blue*), 2 (*yellow*), and 3 (*red*).

There is no high-resolution structure of human P-gp. A homology model based on the crystal structure of mouse P-gp (16) is shown in Fig. 1*A*. The 1280 amino acids are organized into two nucleotide-binding domains (NBDs) and two TM and TMD. Each TMD contains 6 TM segments that extend into the cytoplasm to form ICLs that connect the NBDs to the TMDs. The TMDs interlink (TMs 1, 2, 3, 6, 10, and 11 on one side and TMs 7, 8, 9, 12, 4, and 5 on the other side) to form two divergent wings that attach to NBD1 or NBD2. The protein alternates between open and closed conformations during the predicted catalytic cycle (9). P-gp is shown in an open conformation with the NBDs apart and the drug-binding chamber (pocket surrounded by the TM segments) closed to the outside. In the closed conformation, the NBDs come close together in a head-to-tail orientation to form a pair of ATP-binding sites at their interface and the drug-binding pocket is open to the outside.

McCormick *et al.* (9) predicted that tariquidar could bind at a site composed of ICL2 and ICL3 (site 1) or to one of two sites (sites 2 and 3) located within the TM segments of the TMDs (Fig. 1*B*). Sites 1 and 2 are in one wing (attached to NBD2), whereas site 3 is in the lipid layer between the TM segments of both wings. One approach that could be used to identify domain interfaces affected by substrate binding is cysteine cross-linking protection assays. Cross-linking of cysteines can be readily detected because a disulfide bond between different domains causes the protein to migrate slower on SDS-PAGE gels (34).

The predicted tariquidar-binding sites all lie at interfaces between domains of the two homologous halves of P-gp. Site 1 is at the interface between ICL2 (N-half) and ICL3 (C-half), whereas sites 2 and 3 lie at the interface between TM segments in TMD1 and TMD2. Sites 1 and 2 are on one structural wing, whereas site 3 is located at the interface between the two wings (Fig. 1*B*). Therefore binding of tariquidar at the interface would be predicted to block cysteine cross-linking.

Accordingly, a Cys-less P-gp (35) was used to generate mutants that contained double cysteines at the interfaces involving TM segments of TMD1 and TMD2 (A80C(TM1)/R741C(TM7) (29), I299C(TM5)/F770C(TM8) (36), and T333C(TM6/L975C(TM12) (30), or ICL2 and ICL3 (L175C(ICL1)/N820C(ICL3) (23), A259C(ICL2)/W803C(ICL3) (37), and A266C(ICL2/F1086C(NBD2) (38) (Fig. 1*A*). Mutants containing cross-linkable cysteines between NBD1 and NBD2 (C431(NBD1)/L1176C(NBD2) (39), L531C(NBD1)/C1074(NBD2) (40), and P517C(NBD1)/I1050C(NBD2)) (30) were included as controls. Tariquidar was not expected to inhibit cross-linking between the NBDs as it could rescue a P-gp truncation mutant lacking both NBDs by converting it from a core-glycosylated to a mature form of the mutant (2).

**FIGURE 2.**
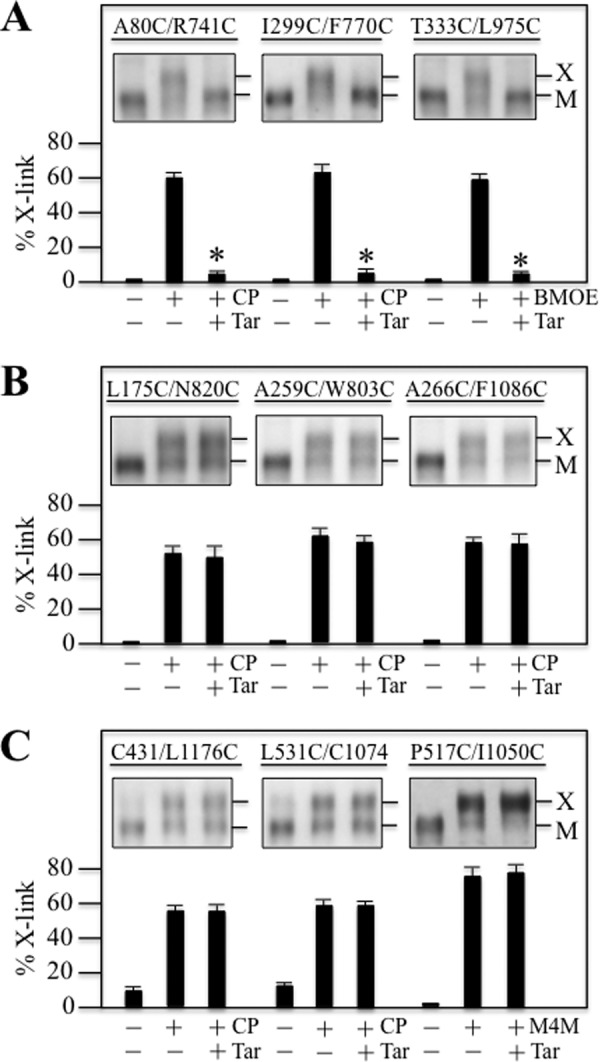
**Tariquidar inhibits cross-linking between cysteines in the TM segments.** Mutants with cysteines in the TM segments (*A*), ICLs (*B*), or NBDs (*C*) were treated in the absence (−) or presence (+) of copper phenanthroline (*CP*), BMOE or *M4M* (BMTS) cross-linker in the absence (−) or presence (+) of tariquidar (*Tar*). Samples were subjected to immunoblot analysis. The locations of cross-linked (*X*) and mature (*M*) P-gps are indicated (*insets*). The amount of cross-linked P-gp (% *X-link*) relative to total P-gp was determined. Each value is the mean ± S.D. (*n* = 3). An *asterisk* indicates significant difference (*p* < 0.001) relative to that cross-linked in the absence of tariquidar.

The cysteine mutants were then expressed in HEK 293 cells. For cross-linking of most mutants, membranes were prepared, incubated with or without tariquidar, and then subjected to cross-linking with copper phenanthroline oxidant. The reactions were stopped by addition of SDS sample buffer containing 25 mm EDTA but no thiol reducing agent and samples subjected to immunoblot analysis. The conditions for cross-linking mutants T333C/L975C and P517C/I1050C, however, were different. Mutant T333C/L975C was cross-linked with BMOE using intact cells because ATP hydrolysis is required for cross-linking (41). Cross-linking of mutant P517C/I1050C was performed on membranes using BMTS cross-linker (30). It was found that 250 nm tariquidar was sufficient to significantly inhibit cross-linking of all three mutants containing double cysteines in the TM segments (A80C(TM1)/R741C(TM7), I299C(TM5)/F770C(TM8), T333C(TM6/L975C(TM12)) (Fig. 2*A*). By contrast, a higher concentration (1000 nm) of tariquidar did not significantly inhibit cross-linking of the ICL (L175C(ICL1)/N820C(ICL3), A259C(ICL2)/W803C(ICL3), A266C(ICL2/F1086C(NBD2)) (Fig. 2*B*) or the NBD (C431(NBD1)/L1176C(NBD2), L531C(NBD1)/C1074(NBD2), and P517C(NBD1)/I1050C(NBD2)) mutants (Fig. 2*C*). These results suggest that tariquidar likely binds to a site at the interface between TMD1 and TMD2 within the lipid bilayer (sites 2 or 3) rather than at the ICL2/ICL3 interface (site 1). This is supported by the recent observation that 500 nm tariquidar efficiently rescued 25 mutants with processing mutations in the ICL2/ICL3 interface (14 mutants in ICL2 and 11 mutants in ICL3) (34).

##### Identification of Arginine Mutations That Inhibit Tariquidar Rescue

Tariquidar acts as a potent corrector to rescue P-gp processing mutants (2). P-gp contains three *N*-glycosylated sites in the extracellular loop that connects TM segments 1 and 2 (Fig. 1*A*). Wild-type P-gp is initially synthesized as a 150-kDa core-glycosylated protein in the endoplasmic reticulum (ER). The protein then leaves the ER and the carbohydrate is modified in the Golgi to yield a 170-kDa mature protein that is then delivered to the cell surface (maturation). The presence of a processing mutation traps P-gp in the ER as a partially folded protease-sensitive core-glycosylated intermediate that is rapidly degraded. Expression of P-gp processing mutants in the presence of drug substrates promotes maturation of the protein to yield functional P-gp at the cell surface (5). Tariquidar acts as a more potent corrector of P-gp processing mutants as concentrations of only about 100 nm are required for rescue (2) compared with concentrations of at least 2000 nm for P-gp substrates such as cyclosporine A, vinblastine, verapamil, and capsaicin (5).

Arginine mutagenesis has been a particularly useful approach to probe the drug-binding pocket structure of P-gp, orientation of the TM segments, and interactions with drug substrates (43, 44). The rationale was that one face of the TM segments of P-gp surround an aqueous chamber (45), whereas the other face is in contact with the surrounding lipid. Insertion of a highly charged arginine side chain (p*K_a_* about 12.5) into a lipid face destabilizes a TM segment because of the large free energy barrier of about 17 kcal/mol for exposing it to lipid hydrocarbon (46). Therefore, insertion of an arginine side chain into the aqueous face of a TM segment would not cause destabilization. Destabilization of a P-gp TM segment is readily detectable because it inhibits P-gp maturation. Arginine mutagenesis enabled us to map the orientation of the 12 TM segments (44) and identify the correct structural model of P-gp (14).

**FIGURE 3.**
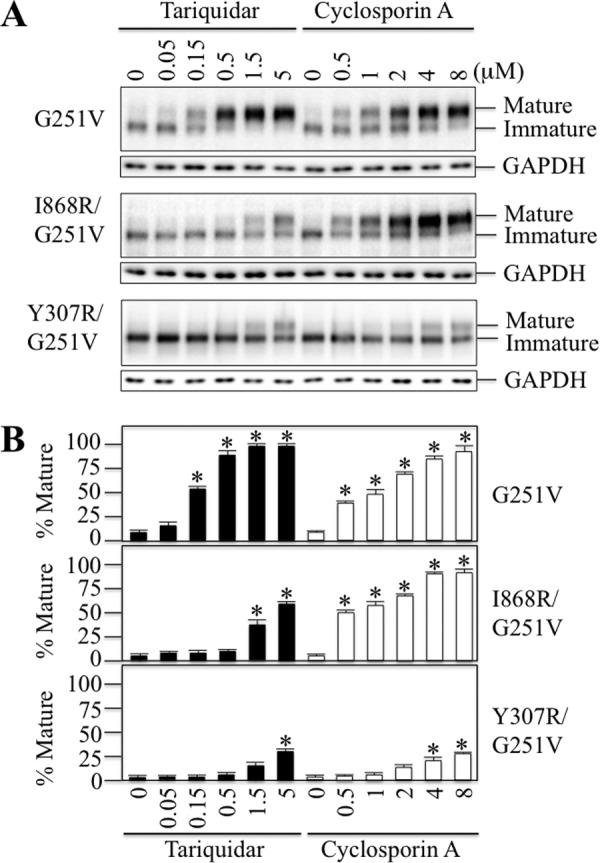
**Arginine residues inhibit rescue of a G251V processing mutant with tariquidar and cyclosporine A.** *A,* whole cell SDS extracts of cells expressing P-gp processing mutant G251V, G251V/I868R, or G251V/Y307R in the presence of various concentrations of tariquidar or cyclosporine A were subjected to immunoblot analysis. The positions of mature and immature P-gps are indicated. *B,* the level of mature P-gp relative to total was determined. Each value is the mean ± S.D. (*n* = 3–5). An *asterisk* indicates significant difference (*p* < 0.001) relative to samples incubated in the absence of drugs.

P-gp substrates are hydrophobic. Insertion of a highly charged arginine side chain into a binding site of a P-gp processing mutant would inhibit the ability of a drug substrate to promote maturation. We have used the G251V processing mutant for arginine mutagenesis and drug rescue assays because the G251V mutation is outside of the drug-binding pocket (located in ICL2) and it yields the 150-kDa immature form of P-gp as the major product when expressed in the absence of drug substrates (44). Drug substrates promote maturation of G251V to yield 170-kDa mature P-gp as the major product. Rescue of G251V could be blocked by the presence of an arginine side chain. For example, we showed that an M69R mutation specifically blocked rescue of a P-gp G251V processing mutant with verapamil but not rescue with cyclosporine A, vinblastine, or rhodamine B (43). Accordingly, we screened the rescue of 223 G251V processing mutants in which each position in the TM segment was replaced with arginine. HEK 293 cells were transfected with each mutant and expressed for 16 h in the absence or presence of 0.5 μm tariquidar. This concentration of tariquidar was chosen because there was nearly complete rescue of the G251V parent (Fig. 3). If an arginine mutant was not rescued with tariquidar, it was tested for rescue with cyclosporine A to check if the arginine specifically inhibited rescue with tariquidar. Cyclosporine A was found to act as a more potent corrector compared with other drug substrates (5).

Two examples of arginine mutations that inhibited rescue of G251V are shown in Fig. 3. The I868R(TM10) mutation inhibited maturation as 10-fold higher levels of tariquidar (5 μm) were required to promote maturation of the 150-kDa protein to yield mature P-gp as the major product. Rescue with cyclosporine A, however, was similar to the G251V parent. By contrast, the Y307R mutation inhibited rescue with both tariquidar and cyclosporine A.

**FIGURE 4.**
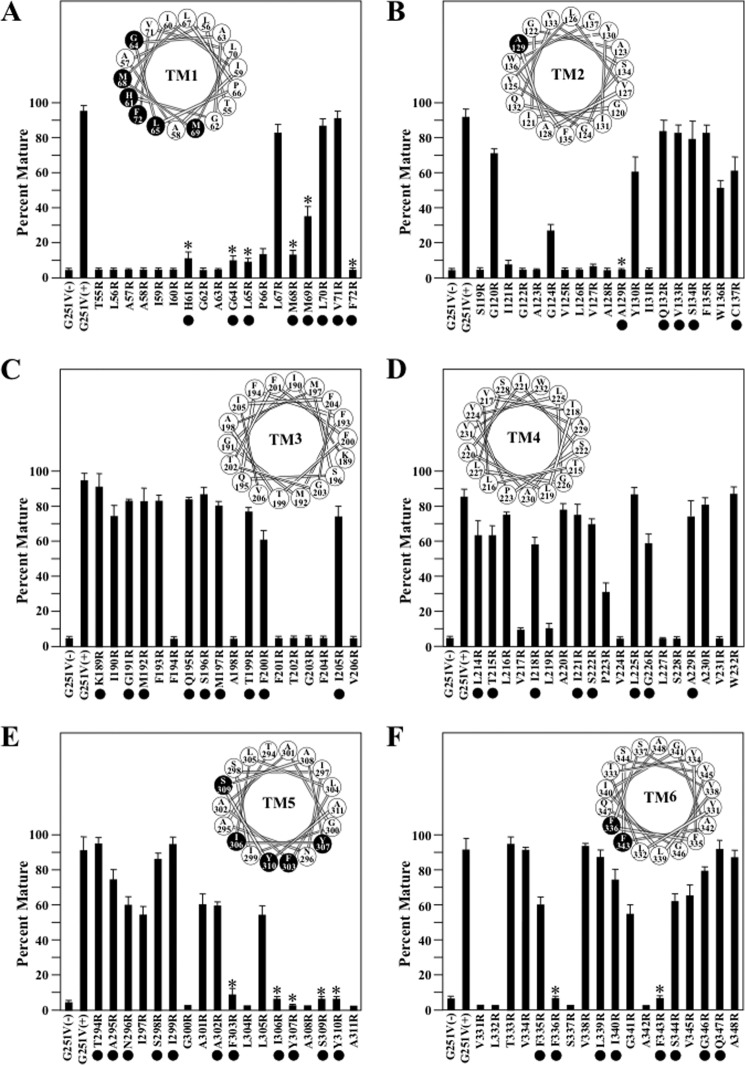
**Rescue of G251V arginine mutants in TMs 1–6 with tariquidar.** Whole cell SDS extracts of cells expressing the G251V mutants containing arginines at various positions in predicted TM1 (*A*), TM2 (*B*), TM3 (*C*), TM4 (*D*), TM5 (*E*), or TM6 (*F*) in the presence of 0.5 μm tariquidar or the G251V parent in the absence (−) or presence (+) of tariquidar were subjected to immunoblot analysis. The amount of mature P-gp (170-kDa protein) relative to total (mature 170-kDa plus immature 150-kDa protein) (*Percent Mature*) was quantified. Each value is the mean ± S.D. (*n* = 3–5). The *black dots* identify residues predicted to line the drug-binding pocket shown in Fig. 5. An *asterisk* indicates a significant decrease (*p* < 0.001) relative to the G251V parent grown in the presence of tariquidar. The *insets* show the positions of the residues in the TM segments when arranged as α-helical wheels. Arginine mutations predicted to line the drug-binding pocket and show significant difference relative to the G251V parent are shown as *black-filled circles*.

**FIGURE 5.**
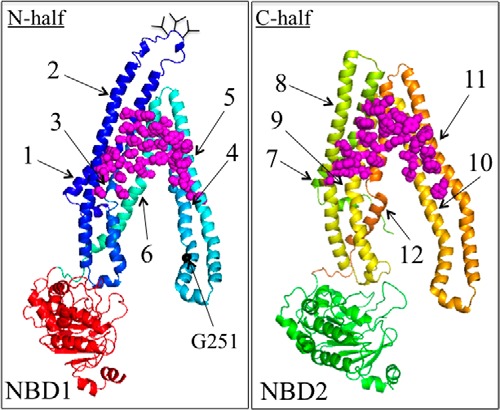
**Location of residues predicted to line the drug-binding pocket.** The two halves of the P-gp model shown in Fig. 1*A* are shown in separate panels. The C-terminal half (C-half) has been rotated to view the residues facing the drug-binding pocket (side chains shown as *magenta spheres*). The TM segments are numbered.

The effects of arginines introduced into the TM segments of TMD1 on rescue of G251V with 0.5 μm tariquidar are shown in Fig. 4. Our goal was to identify arginines that protrude into the drug-binding pocket and could inhibit tariquidar binding and therefore rescue of the mutant. A potential problem is that arginines may inhibit tariquidar rescue since they disrupt protein folding and rescue because they are located at a lipid or TM segment interface. Therefore, we focused on residues predicted to line the drug-binding pocket (Fig. 5) and identify those that inhibit rescue with tariquidar.

In the N-terminal TMD1 domain, the largest number of arginine mutations predicted to line the drug-binding pocket that inhibited tariquidar rescue were located in TM1 (H61R, G64R, L65R, M68R, M69R, and F72R) and TM5 (F303R, I306R, Y307R, S309R, and Y310R) (Fig. 4, *A* and *E*). One arginine mutation predicted to line the drug-binding pocket inhibited rescue in TM2 (A129R) (Fig. 4*B*) and two arginines predicted to line the drug-binding pocket in TM6 (F336R and F343R) were found to inhibit tariquidar rescue (Fig. 4*F*). No arginines predicted to line the drug-binding pocket were found to block tariquidar rescue in TM segments 3 or 4 (Fig. 4, *C* and *D*).

**FIGURE 6.**
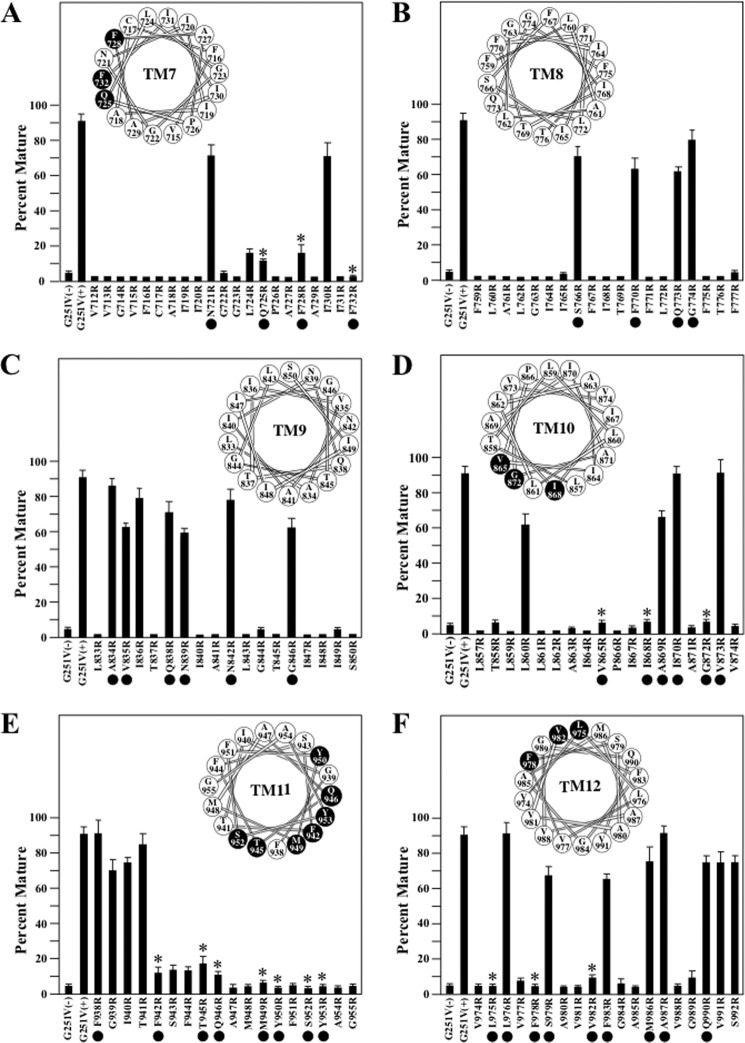
**Rescue of G251V arginine mutants in TMs 7–12 with tariquidar.** Whole cell SDS extracts of cells expressing the G251V mutants containing arginines at various positions in predicted TM7 (*A*), TM8 (*B*), TM9 (*C*), TM10 (*D*), TM11 (*E*), or TM12 (*F*) in the presence of 0.5 μm tariquidar or the G251V parent in the absence (−) or presence (+) of tariquidar were subjected to immunoblot analysis. The amount of mature P-gp (170-kDa protein) relative to total (mature 170-kDa plus immature 150-kDa protein) (*Percent Mature*) was quantified. Each value is the mean ± S.D. (*n* = 3–5). The *black dots* identify residues predicted to line the drug-binding pocket shown in Fig. 5. An *asterisk* indicates a significant decrease (*p* < 0.001) relative to the G251V parent grown with tariquidar. The positions of the residues in the TM segments when arranged as α-helical wheels are shown in the *insets*. The *black-filled circles* represent arginine mutations predicted to line the drug-binding pocket and show significant difference relative to the G251V parent.

Tariquidar rescue of the G251V/arginine mutants in TMD2 are shown in Fig. 6. In TMD2, many arginine mutants predicted to line the drug-binding pocket and inhibited tariquidar rescue were identified. These were in TM7 (Q725R, F728R, and F732R) (Fig. 6*A*), TM10 (V865R, I868R, and G872R) (Fig. 6*D*), TM11 (F942R, T945R, Q946R, M949R, Y950R, and Y953R) (Fig. 6*E*), and TM12 (L975R, F978R, and V982R) (Fig. 6*F*). No arginines predicted to line the drug-binding pocket were found to block tariquidar rescue in TM segments 8 or 9 (Fig. 6, *B* and *C*).

**FIGURE 7.**
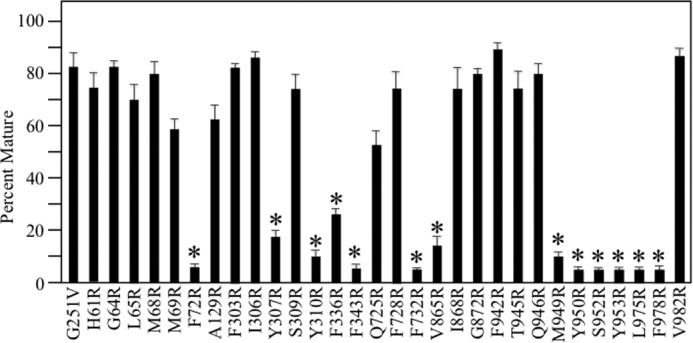
**Rescue of G251V arginine mutants with cyclosporine A.** Cells expressing the G251V parent or G251V/arginine mutants that could not be rescued with tariquidar (identified as *black circles* in the α-helical wheels shown in Figs. 4 and 6) were expressed in the presence of 5 μm cyclosporine A. Whole cell SDS extracts were subjected to immunoblot analysis. The amount of mature P-gp (170-kDa protein) relative to total (mature 170-kDa plus immature 150-kDa protein) (*Percent Mature*) was quantified. Each value is the mean ± S.D. (*n* = 3–5). An *asterisk* indicates a significant difference (*p* < 0.001) relative to the G251V parent grown in the presence of cyclosporine A.

A total of 30 arginine mutants predicted to line the drug-binding pocket inhibited rescue of G251V with tariquidar (Figs. 4 and 6). Fig. 3 showed that the Y307R(TM5) mutation but not I868R(TM10) also inhibited rescue with cyclosporine A. Therefore, do other arginine mutations inhibit rescue of G251V with cyclosporine A? To address this question, we tested for rescue of the other 28 arginine mutants with 5 μm cyclosporine A as this concentration showed robust rescue of the G251V parent (Fig. 3). It was found that 16 of the 28 mutants resembled the G251V/I868R mutant as expression in the presence of 5 μm cyclosporine A yielded mature P-gp as the major product in TM1 (H61R, G64R, L65R, M68R, and M69R), TM5 (F303R, I306R, and S309R), TM7 (Q725R and F728R), TM10 (I868R and G872R), TM11 (F942R, T945R, and Q946R), and TM12 (V982R) (Fig. 7). The other 12 mutants in TM1 (F72R), TM5 (Y307R and Y310R), TM6 (F336R and F343R), TM7 (F732R), TM10 (V865R), TM11 (M949R, Y950R, S952R, and Y953R), and TM12 (L975R and F978R) were not rescued by cyclosporine A (Fig. 7). The results suggest that there may be partial overlap of the tariquidar and cyclosporine A binding sites as predicted by Chufan *et al.* (8).

##### Arginine Mutations Inhibit Tariquidar-stimulated ATPase Activity

A potential disadvantage of the tariquidar-rescue assay is that it involves rescue of a P-gp processing mutant. Would the arginine mutations disrupt tariquidar interactions in a wild-type background? To address this question, we assayed for tariquidar-stimulated ATPase activity of purified arginine mutants. Drugs modulate P-gp ATPase activity (47) and the level of activation or reduction in basal ATPase activity is highly dependent on the composition of the lipid (48). Tariquidar can act as a stimulator or inhibitor of human P-gp ATPase activity depending on its lipid environment. For example, we^3^ and others (8) found that tariquidar and some drug substrates inhibited human P-gp ATPase activity when it is expressed in insect Sf9 cells as P-gp shows a high basal ATPase activity when incorporated into Sf9 lipids. P-gp shows a very low basal ATPase activity when incorporated into sheep brain lipid (49) and its ATPase activity is activated by tariquidar (30).

Accordingly, each of the 30 arginine residues (Figs. 4 and 6) was introduced into a histidine-tagged wild-type background. The histidine-tagged mutants were expressed in HEK 293 cells and P-gp isolated by nickel-chelate chromatography (26). The mutants were expressed at 30 °C in the presence of 10 μm cyclosporine A to promote maturation. All of the arginine mutants in the wild-type background yielded mature P-gp as the major product when expressed at low temperature (30 °C) in the presence of cyclosporine A (data not shown). P-gp processing mutants resemble processing mutants of its CFTR sister protein as expression at low temperature (30 °C) promotes maturation of the mutants (50).

**FIGURE 8.**
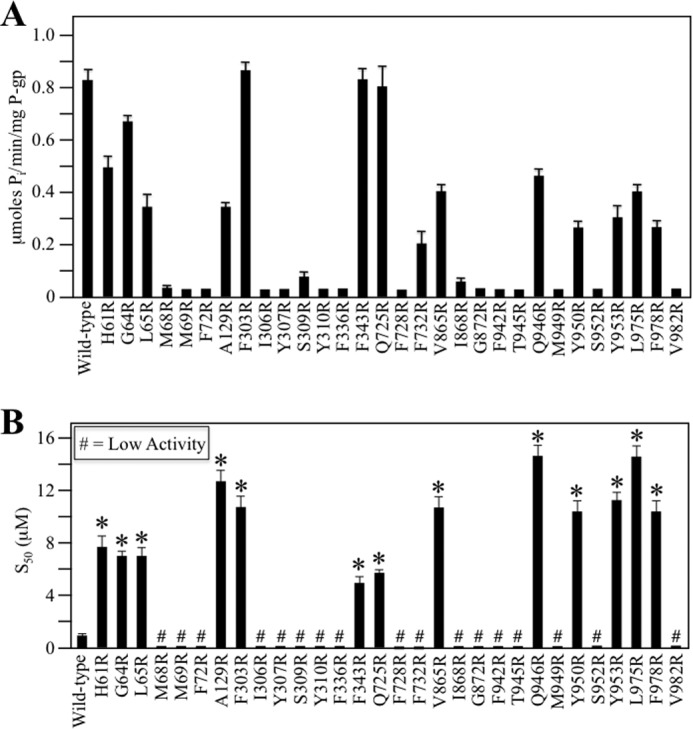
**Mutants defective in tariquidar rescue show reduced tariquidar-stimulated ATPase activity.** Histidine-tagged wild-type or mutant P-gps (in wild-type background) containing arginines defective in tariquidar rescue (identified as *black circles* in the α-helical wheels shown in Figs. 4 and 6) were expressed in HEK 293 cells and isolated by nickel-chelate chromatography. The isolated P-gps were mixed with lipid and ATPase activities were measured in the presence of a saturating concentration of tariquidar (60 μm) to determine maximal activity (*A*) or in the presence of various concentrations of tariquidar to determine the concentration required to stimulate ATPase activity by 50% (S_50_) (*B*) are shown. Each value is the mean ± S.D. (*n* = 3). An *asterisk* in *B* indicates a significant difference (*p* < 0.001) relative to wild-type P-gp.

The isolated mutant P-gps were mixed with lipid and assayed for ATPase activity in the presence of various concentrations of tariquidar. Wild-type P-gp exhibited basal ATPase activity of about 0.1 μm P_i_/min/mg of P-gp. The ATPase activity was then measured in the presence of various concentrations of tariquidar (0–60 μm). Wild-type P-gp ATPase activity was maximally stimulated about 8-fold (Fig. 8*A*) and had a half-maximal stimulation (S_50_) at a concentration of about 0.8 μm tariquidar (Fig. 8*B*). Seventeen of the 30 G251V/arginine mutants (M68R, M69R, and F72R in TM1; I306R, Y307R, S309R, and Y310R in TM5; F336R in TM6; F728R and F732R in TM7; I868R and G872R in TM10; F942R, T945R, M949R, and S952R in TM11; and V982R in TM12) that could not be rescued with tariquidar showed little or no stimulation of ATPase activity with tariquidar (Fig. 8*A*). Mutants L65R(TM1), A129R(TM2), F732R(TM7), Y950R(TM11), Y953R(TM11), and F978R(TM12) showed partial activity (about 25–50% of wild-type activity), whereas the maximal tariquidar-stimulated ATPase activity of mutants H61R(TM1), G64R(TM1), F303R(TM5), F343R(TM6), Q725R(TM7), V865R(TM10, Q946R(TM11), and L975R(TM12) was about 50–100% of wild-type enzyme. All of the active arginine mutants, however, had S_50_ concentrations that were at least 5-fold higher than wild-type P-gp (Fig. 8*B*).

**FIGURE 9.**
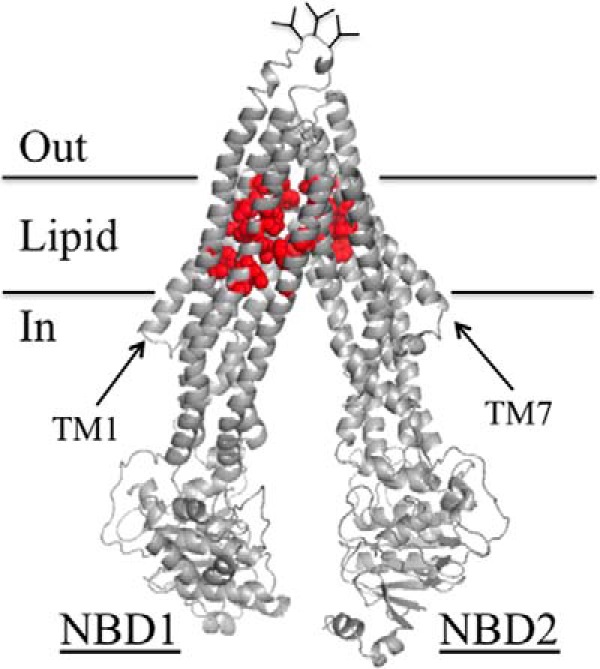
**Location of the arginine mutations that inhibit tariquidar rescue of misprocessed mutants and tariquidar-stimulated ATPase activity.** Predicted structure of human P-gp in the open conformation as described in the legend to Fig. 1. The *red balls* show the positions of residues in the TM segments (His^61^, Gly^64^, Leu^65^, Met^68^, Met^69^, Phe^72^, Ala^129^, Phe^303^, Ile^306^, Tyr^307^, Ser^309^, Tyr^310^, Phe^336^, Phe^343^, Gln^725^, Phe^728^, Phe^732^, Val^865^, Ile^868^, Gly^872^, Phe^942^, Thr^945^, Gln^946^, Met^949^, Tyr^950^, Ser^952^, Tyr^953^, Leu^975^, Phe^978^, and Val^982^) that when changed to arginine significantly reduced both arginine rescue (Figs. 4 and 6) and tariquidar-stimulated ATPase activity (Fig. 8).

The decrease in the affinities of these mutants for tariquidar in their ATPase activities was consistent with their inability to be rescued with tariquidar (Figs. 4 and 6). These results indicate that the arginine side chain in these 30 mutants perturbs P-gp/tariquidar interactions. The locations of these residues are shown in Fig. 9. They overlap with many residues predicted *in silico* to be present in site 3 (9) of the tariquidar-binding sites (Fig. 1*B*)

##### Tariquidar Promotes Maturation of TMD1 into a Protease-resistant Conformation

Rescue of processing mutants by tariquidar and cyclosporine A showed differences because many mutants containing processing mutations outside the drug-binding pocket could only be rescued with tariquidar (2). Tariquidar was about 10 times more potent than cyclosporine A in rescuing most processing mutants. This suggested that both compounds might rescue processing mutants by different mechanisms.

One possibility is that rescue by tariquidar resembles the rescue of CFTR processing mutants with VX-809 (22). Corrector VX-809 is the most effective pharmacological chaperone to repair CFTR processing mutants (51). Its mechanism of rescue involves stabilization of TMD1 to yield a protease-resistant protein (22). By contrast, the mechanism of cyclosporine A rescue of P-gp mutants involves stabilization of TMD2 (27). Cyclosporine A did not promote maturation of P-gp TMD1 expressed as a separate protein or convert it into a protease-resistant conformation (27).

**FIGURE 10.**
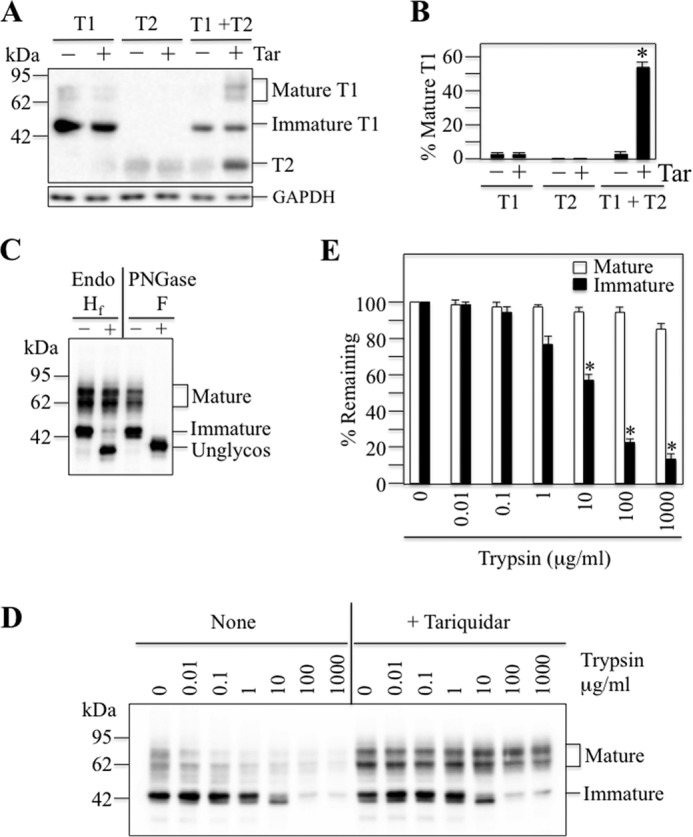
**Tariquidar stabilizes TMD1.** *A,* A52-tagged TMD1 (T1) (residues 1–379), A52-tagged TMD2 (T2) (residues 681–1025), or both A52-tagged TMD1 plus A52-tagged TMD2 (T1 + T2) were expressed in the absence (−) or presence (+) of 1 μm tariquidar (*Tar*). Whole cell SDS extracts were subjected to immunoblot analysis. The locations of TMD2 (T2) and mature and immature forms of TMD1 (T1) are indicated. *B,* the amount of mature TMD1 (T1) relative to total (mature and immature TMD1) (% Mature T1) was quantified. Each value is the mean ± S.D. (*n* = 3–5). An *asterisk* indicates a significant difference (*p* < 0.001) relative to samples grown in the absence of tariquidar. *C*, HEK 293 cells expressing A52-tagged TMD1 plus untagged TMD2 and grown in the presence of tariquidar were treated without (−) or with (+) endoglycosidase H (Endo H_f_) or PNGase F. The reactions were stopped by addition of SDS sample buffer and subjected to immunoblot analysis. The positions of mature, immature, and unglycosylated (Unglycos) forms of TMD1 are indicated. *D,* membranes prepared from cells expressing A52-tagged TMD1 plus untagged TMD2 and grown in the absence (None) or presence (+Tariquidar) of tariquidar were treated with various concentrations of trypsin. The reactions were stopped by addition of trypsin inhibitor and samples were subjected to immunoblot analysis. The positions of mature and immature forms of TMD1 are indicated. *E,* the levels of mature (*filled bars*) or immature (*unfilled bars*) forms of TMD1 were determined and the amount remaining (% *Remaining*) after treatment with various levels of trypsin was compared with the untreated control. An *asterisk* indicates a significant difference (*p* < 0.001; *n* = 3) relative to that without trypsin.

Therefore, we tested if tariquidar would promote maturation or stabilize P-gp TMD1. HEK 293 cells were transfected with A52-tagged TMD1 (residues 1–379), A52-tagged TMD2 (residues 681–1025), or A52-tagged TMD1 plus A52-tagged TMD2 and grown in the absence or presence of tariquidar. Whole cell SDS extracts were then subjected to immunoblot analysis (Fig. 10). Maturation of TMD1 in the presence of tariquidar occurred only when TMD1 was co-expressed with TMD2 (Fig. 10, *A* and *B*).

A52-tagged TMD1 together with untagged TMD2 were co-expressed in HEK 293 cells in the presence of tariquidar. The cells were solubilized and treated with endoglycosidases H (H_f_) and F (PNGase F). Samples were then subjected to immunoblot analysis. Fig. 10*C* shows that tariquidar promoted maturation of TMD1 such that the mature product was resistant to endoglycosidase H but not to endoglycosidase F. By contrast, the immature form of TMD1 was sensitive to both endoglycosidases. These results indicate that the immature product is in the ER, whereas the mature form of TMD1 has traversed the Golgi apparatus.

We previously showed that P-gp folds into a more compact conformation when it leaves the ER such that it becomes more resistant to protease digestion (27). Therefore, we tested whether the mature form of TMD1 also became resistant to protease digestion. Membranes were prepared from HEK 293 cells co-expressing A52-tagged TMD1 and untagged TMD2 and expressed in the absence or presence of tariquidar. Samples of the membranes were then treated with various concentrations of TPCK-trypsin for 5 min at 20 °C. It was found that the mature form of TMD1 was over 100-fold more resistant to trypsin compared with the immature protein (Fig. 10, *D* and *E*). About 10 μg/ml of trypsin was required to reduce the amount of immature TMD1 by 50%, whereas mature TMD1 remained resistant to trypsin at the highest concentration tested (1000 μg/ml) (Fig. 10*E*).

The results showed that tariquidar differed from cyclosporine A. We previously reported that cyclosporine A did not stabilize TMD1 (27). Here we showed that tariquidar promoted maturation of the TMD1 protein into a protease-resistant conformation.

## Discussion

**FIGURE 11.**
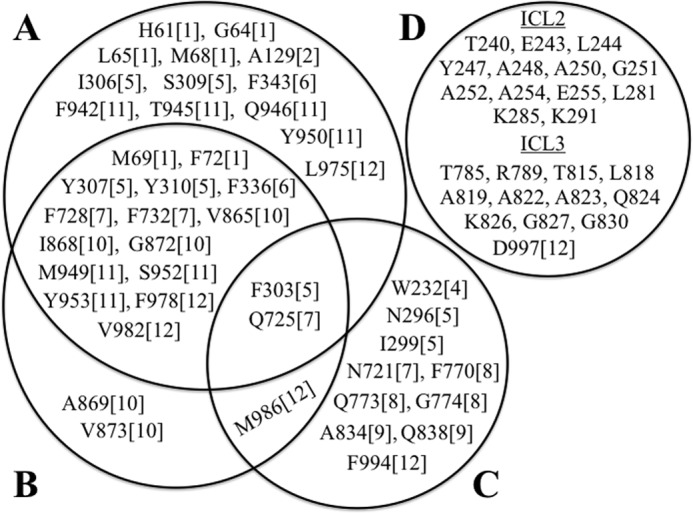
**Residues predicted to lie close to the tariquidar-binding site by arginine mutagenesis and molecular docking studies.** Residues predicted to lie close to the tariquidar-binding site by arginine mutagenesis (*A*, this study) or by *in silico* molecular docking (9) to site 1 (*D*), site 2 (*C*), and site 3 (*B*) are shown. The *number in the bracket* indicates the TM segment where the residue is located.

Through arginine mutagenesis we identified 30 positions (Fig. 11*A*) in the predicted drug-binding pocket where arginines caused major disruptions in the ability of tariquidar to rescue processing mutants or activate ATPase activity. McCormick *et al.* (9) used molecular docking studies to identify two potential tariquidar-binding sites in the predicted drug-binding domain (sites 2 and 3). When we compared the results of the arginine mutagenesis studies to the molecular docking predictions, it was observed that 17 of the 20 residues predicted to lie close to the tariquidar-binding site in site 3 showed overlap (Fig. 11*B*). By contrast, only 2 of 13 residues of the predicted site 2 tariquidar-binding site showed overlap with residues identified by arginine mutagenesis (Fig. 11*C*). Therefore, the results of the cross-linking, arginine mutagenesis studies, and molecular docking predictions suggest that predicted site 3 is the likely high affinity-binding site for tariquidar.

Binding of tariquidar to site 3 might help explain the mechanism of its effectiveness as a P-gp corrector and inhibitor. Site 3 is located in the lipid bilayer at the interface between the two wings of P-gp (Fig. 1*B*). Binding of tariquidar at this site would enable it to stabilize both TMDs (enhanced corrector activity) and bring the two wings and NBDs together into a closed conformation that would result in stimulation of ATPase activity but with no efflux activity (inhibitor). This would be consistent with the observation that tariquidar appears to trap P-gp in a closed conformation (2).

We identified 13 additional arginine mutations (H61R, G64R, L65R, and M68R in TM1; A129R in TM2; I306R and S309R in TM5; F343R in TM6; F942R, T945R, Q946R, and Y950R in TM11; and L975R in TM12) (Fig. 11*A*) that were not predicted to lie within 4.5-Å of the predicted site 3 tariquidar-binding site (9). Chufan *et al.* (8) also used the corrected mouse crystal structure as a template to perform molecular docking studies of tariquidar to a homology model of human P-gp. Chufan *et al.* (8) identified 14 residues that were identical to those in site 3 (20 residues) predicted by McCormick *et al.* (9). Chufan *et al.* (8) also identified three other different residues (Leu^65^ (TM1), Gln^946^ (TM11), and Leu^975^ (TM12)) that were in agreement with our results. Because there is no high-resolution structure of human P-gp, the different residues identified to be involved in tariquidar binding by the two *in silico* methods could be due to different algorithms and programs used to generate the homology models of human P-gp for the docking studies. It could also be due to the scoring function chosen for best docking (52). The lowest binding energy score is often chosen in docking studies, but this might not be the correct pose that reflects actual drug binding (52).

A reason for the identification of additional residues in our study that were not predicted by the *in silico* docking studies was that some arginines might have perturbed the structure of TM segments. For example, 8 of the 13 arginines with reduced tariquidar interactions are located in TM1 and TM11 (Fig. 11*A*). Cysteine cross-linking studies showed that TM segments 1 and 11 in human P-gp were adjacent to each other and that interactions between TM segments 1 and 11 were altered during the catalytic cycle by ATP hydrolysis (53). In addition, suppressor mutations in TM1 were found to increase folding and maturation of P-gp processing mutations by promoting interactions between TM segments 1 and 11 (43). Therefore, arginines predicted to lie outside of the tariquidar-binding site in TM1 and TM11 in the docking studies (H61R, G64R, L65R, and M68 in TM1; F942R, T945R, Q946R, and Y950R in TM11) might alter interactions between TM1 and TM11.

Finally, another explanation for differences between the arginine mutagenesis and *in silico* docking studies (8, 9) is that there is evidence that P-gp recognizes drug substrates by an induced-fit mechanism (54). For example, binding of drug substrates was found to change the cross-linking pattern between TM segments 6/11 and 6/12 (54). Changes in the cross-linking pattern suggested that binding of a drug could induce repacking or rotation of the TM segments (54).

This study shows that *in silico* docking studies are potentially valuable tools in aiding the mapping of binding site(s) of a drug substrate/inhibitor by biochemical methods. The results in this study suggest that tariquidar binds to a binding site that is within the TMDs and is consistent with the idea that there is a large flexible drug-binding pocket with the TMDs that can accommodate other drug substrates (16, 55–58). It is unknown why tariquidar is not transported like other drug substrates even though it still stimulates ATPase activity. It is possible that tariquidar is not transported because it binds with a very high affinity for P-gp (59) to activate ATPase activity but is not released during the catalytic cycle (4) or it could simply move from a high-affinity site to a low-affinity site or to a silent non-transported site and bound in a different orientation within the drug-binding pocket during the catalytic cycle. This could simply involve a change in the orientation of tariquidar in the drug-binding pocket. This behavior is not unusual as labeling studies of P-gp cysteine mutants with thiol-reactive substrates of verapamil (60, 61) or rhodamine B (55) could covalently attach to different residues within the drug-binding pocket and still activate ATPase activity (31, 60–62). Therefore it appears that drug substrates could bind in different orientations within the drug-binding pocket.

Tariquidar is a transport substrate of BCRP, another ABC drug pump. BCRP functions as a dimer with each monomer consisting of one NBD and a TMD made up of 6 TM segments (63). BCRP is different from P-gp because it has a much lower affinity for tariquidar (4). In ATPase assays performed in membrane preparations overexpressing the proteins, a 50% change in ATPase activity was observed with about 5 nm tariquidar for P-gp (59). BCRP required about 30-fold higher levels (about 150 nm) of tariquidar to activate ATPase activity by 50% (4). It should be noted that we required about 100-fold higher levels of tariquidar in the ATPase assays (about 500–1000 nm) because the presence of detergent increases the apparent affinity of drug substrates for P-gp by 100-fold or more (64).

We found that the mechanism of rescue with tariquidar was different from that with cyclosporine A because it could promote maturation and stabilize TMD1 (Fig. 10). Conversion from a protease-sensitive conformation to a protease-resistant conformation appears to be an important step in P-gp maturation (65). Processing mutations trap the protein in the ER in a loosely folded conformation with incomplete packing of the TM segments. Drug substrates promote packing of the TM segments to induce the protein to complete the folding process.

CFTR shows a similar mechanism. CF mutations like ΔF508 inhibit maturation by trapping the protein in a protease-resistant pre-folded state (66) with incomplete packing of the TM segments (67). Specific correctors can bind to the protein (68) in the ER (69) to promote maturation. Unfortunately, the correctors identified to date only promote maturation of a small fraction of the molecules, and hence are poor therapeutic candidates. Efficient correctors also need to be developed for other ABC processing mutants that cause disease such as ABCG2 (gout), ABCB4/ABCB11 (progressive familial intrahepatic cholestasis), ABCA1 (Tangier disease), ABCC2 (Dubin-Johnson syndrome), ABCC6 (pseudoxanthoma elasticum), and ABCC8 (hyperinsulinimic hypoglycemia of infancy).

Identification of a universal target to repair ABC processing mutants would aid in the development of correctors for different diseases. Our studies on CFTR and P-gp suggest that TMD1 would be an attractive target because the best correctors for P-gp (tariquidar) and CFTR (VX-809) stabilized the TMD1 domain. Our tariquidar mapping studies suggest that molecular docking studies can be a useful approach to identify a target site.

## Author Contributions

Both authors designed and conducted the experiments. Both authors analyzed the data, wrote the article, and approve of the version to be published.

## Footnotes

3 T. W. Loo and D. M. Clarke, unpublished data.

## Footnotes

2 The abbreviations used are:

ABC: ATP-binding cassette
BMOE: bismaleimidoethane
CFTR: cystic fibrosis transmembrane conductance regulator
BMTS: 1,4-butanediyl bismethanethiosulfonate
P-gp: P-glycoprotein
NBD: nucleotide-binding domain
TM: transmembrane
TMD: transmembrane domain
ICL: intracellular loop
BES: *N*,*N*-bis(2-hydroxyethyl)-2-aminoethanesulfonic acid
BMOE: bismaleimidoethane
PNGase F: peptide *N*-glycosidase F
TPCK: l-1-tosylamido-2-phenylethyl chloromethyl ketone
ER: endoplasmic reticulum.

## References

[1] Borst P., and Elferink R. O. (2002) Mammalian abc transporters in health and disease. Annu. Rev. Biochem. 71, 537–592 12045106 10.1146/annurev.biochem.71.102301.093055

[2] Loo T. W., and Clarke D. M. (2014) Tariquidar inhibits P-glycoprotein drug efflux but activates ATPase activity by blocking transition to an open conformation. Biochem. Pharmacol. 92, 558–566 25456855 10.1016/j.bcp.2014.10.006

[3] Wagner C. C., Bauer M., Karch R., Feurstein T., Kopp S., Chiba P., Kletter K., Löscher W., Müller M., Zeitlinger M., and Langer O. (2009) A pilot study to assess the efficacy of tariquidar to inhibit P-glycoprotein at the human blood-brain barrier with (*R*)-11C-verapamil and PET. J. Nucl. Med. 50, 1954–1961 19910428 10.2967/jnumed.109.063289 PMC3690436

[4] Kannan P., Telu S., Shukla S., Ambudkar S.V., Pike V.W., Halldin C., Gottesman M.M., Innis R.B., and Hall M.D. (2011) The “specific” P-glycoprotein inhibitor tariquidar is also a substrate and an inhibitor for breast cancer resistance protein (BCRP/ABCG2). ACS Chem. Neurosci. 2, 82–89 22778859 10.1021/cn100078a PMC3369725

[5] Loo T. W., and Clarke D. M. (1997) Correction of defective protein kinesis of human P-glycoprotein mutants by substrates and modulators. J. Biol. Chem. 272, 709–712 8995353 10.1074/jbc.272.2.709

[6] Loo T. W., Bartlett M. C., and Clarke D. M. (2013) Bithiazole correctors rescue CFTR mutants by two different mechanisms. Biochemistry 52, 5161–5163 23865422 10.1021/bi4008758 PMC3737597

[7] Pajeva I. K., Hanl M., and Wiese M. (2013) Protein contacts and ligand binding in the inward-facing model of human P-glycoprotein. ChemMedChem 8, 748–762 23564544 10.1002/cmdc.201200491

[8] Chufan E. E., Kapoor K., Sim H. M., Singh S., Talele T. T., Durell S. R., and Ambudkar S. V. (2013) Multiple transport-active binding sites are available for a single substrate on human P-glycoprotein (ABCB1). PLoS ONE 8, e82463 24349290 10.1371/journal.pone.0082463 PMC3857843

[9] McCormick J. W., Vogel P. D., and Wise J. G. (2015) Multiple drug transport pathways through human P-glycoprotein. Biochemistry 54, 4374–4390 26125482 10.1021/acs.biochem.5b00018 PMC4527178

[10] Jara G. E., Vera D. M., and Pierini A. B. (2013) Binding of modulators to mouse and human multidrug resistance P-glycoprotein: a computational study. J. Mol. Graph. Model. 46, 10–21 24095875 10.1016/j.jmgm.2013.09.001

[11] Pajeva I. K., Sterz K., Christlieb M., Steggemann K., Marighetti F., and Wiese M. (2013) Interactions of the multidrug resistance modulators tariquidar and elacridar and their analogues with P-glycoprotein. ChemMedChem 8, 1701–1713 23943604 10.1002/cmdc.201300233

[12] Prajapati R., and Sangamwar A. T. (2014) Translocation mechanism of P-glycoprotein and conformational changes occurring at drug-binding site: insights from multi-targeted molecular dynamics. Biochim. Biophys. Acta 1838, 2882–2898 25068895 10.1016/j.bbamem.2014.07.018

[13] Kong L. L., Zhuang X. M., Yang H. Y., Yuan M., Xu L., and Li H. (2015) Inhibition of P-glycoprotein gene expression and function enhances triptolide-induced hepatotoxicity in mice. Sci. Rep. 5, 11747 26134275 10.1038/srep11747 PMC4488747

[14] Loo T. W., and Clarke D. M. (2013) Drug rescue distinguishes between different structural models of human P-glycoprotein. Biochemistry 52, 7167–7169 24083983 10.1021/bi401269m PMC3798097

[15] Loo T. W., and Clarke D. M. (2013) A salt bridge in intracellular loop 2 is essential for folding of human P-glycoprotein. Biochemistry 52, 3194–3196 23634976 10.1021/bi400425k PMC3656768

[16] Li J., Jaimes K. F., and Aller S. G. (2014) Refined structures of mouse P-glycoprotein. Protein Sci. 23, 34–46 24155053 10.1002/pro.2387 PMC3892297

[17] Ward A. B., Szewczyk P., Grimard V., Lee C. W., Martinez L., Doshi R., Caya A., Villaluz M., Pardon E., Cregger C., Swartz D. J., Falson P. G., Urbatsch I. L., Govaerts C., Steyaert J., and Chang G. (2013) Structures of P-glycoprotein reveal its conformational flexibility and an epitope on the nucleotide-binding domain. Proc. Natl. Acad. Sci. U.S.A. 110, 13386–13391 23901103 10.1073/pnas.1309275110 PMC3746859

[18] Clarke D. M., Loo T. W., Inesi G., and MacLennan D. H. (1989) Location of high affinity Ca^2+^-binding sites within the predicted transmembrane domain of the sarcoplasmic reticulum Ca^2+^-ATPase. Nature 339, 476–478 2524669 10.1038/339476a0

[19] Loo T. W., and Clarke D. M. (1993) Functional consequences of proline mutations in the predicted transmembrane domain of P-glycoprotein. J. Biol. Chem. 268, 3143–3149 8094081

[20] Loo T. W., and Clarke D. M. (1993) Functional consequences of phenylalanine mutations in the predicted transmembrane domain of P-glycoprotein. J. Biol. Chem. 268, 19965–19972 8104183

[21] Choi K. H., Chen C. J., Kriegler M., and Roninson I. B. (1988) An altered pattern of cross-resistance in multidrug-resistant human cells results from spontaneous mutations in the *mdr1* (P-glycoprotein) gene. Cell 53, 519–529 2897240 10.1016/0092-8674(88)90568-5

[22] Loo T. W., Bartlett M. C., and Clarke D. M. (2013) Corrector VX-809 stabilizes the first transmembrane domain of CFTR. Biochem. Pharmacol. 86, 612–619 23835419 10.1016/j.bcp.2013.06.028

[23] Loo T. W., Bartlett M. C., and Clarke D. M. (2010) Human P-glycoprotein is active when the two halves are clamped together in the closed conformation. Biochem. Biophys. Res. Commun. 395, 436–440 20394729 10.1016/j.bbrc.2010.04.057

[24] Kunkel T. A. (1985) Rapid and efficient site-specific mutagenesis without phenotypic selection. Proc. Natl. Acad. Sci. U.S.A. 82, 488–492 3881765 10.1073/pnas.82.2.488 PMC397064

[25] Loo T. W., and Clarke D. M. (1994) Functional consequences of glycine mutations in the predicted cytoplasmic loops of P-glycoprotein. J. Biol. Chem. 269, 7243–7248 7907326

[26] Loo T. W., and Clarke D. M. (1995) Rapid purification of human P-glycoprotein mutants expressed transiently in HEK 293 cells by nickel-chelate chromatography and characterization of their drug-stimulated ATPase activities. J. Biol. Chem. 270, 21449–21452 7665554 10.1074/jbc.270.37.21449

[27] Loo T. W., and Clarke D. M. (1998) Superfolding of the partially unfolded core-glycosylated intermediate of human P-glycoprotein into the mature enzyme is promoted by substrate-induced transmembrane domain interactions. J. Biol. Chem. 273, 14671–14674 9614062 10.1074/jbc.273.24.14671

[28] Loo T. W., Bartlett M. C., and Clarke D. M. (2008) Processing mutations disrupt interactions between the nucleotide binding and transmembrane domains of P-glycoprotein and the cystic fibrosis transmembrane conductance regulator (CFTR). J. Biol. Chem. 283, 28190–28197 18708637 10.1074/jbc.M805834200 PMC2661390

[29] Loo T. W., and Clarke D. M. (2014) Cysteines introduced into extracellular loops 1 and 4 of human P-glycoprotein that are close only in the open conformation spontaneously form a disulfide bond that inhibits drug efflux and ATPase activity. J. Biol. Chem. 289, 24749–24758 25053414 10.1074/jbc.M114.583021 PMC4155644

[30] Loo T. W., Bartlett M. C., Detty M. R., and Clarke D. M. (2012) The ATPase activity of the P-glycoprotein drug pump is highly activated when the N-terminal and central regions of the nucleotide-binding domains are linked closely together. J. Biol. Chem. 287, 26806–26816 22700974 10.1074/jbc.M112.376202 PMC3411018

[31] Loo T. W., Bartlett M. C., and Clarke D. M. (2006) Transmembrane segment 7 of human P-glycoprotein forms part of the drug-binding pocket. Biochem. J. 399, 351–359 16813563 10.1042/BJ20060715 PMC1609921

[32] Gorman C. M., Howard B. H., and Reeves R. (1983) Expression of recombinant plasmids in mammalian cells is enhanced by sodium butyrate. Nucleic Acids Res. 11, 7631–7648 6316266 10.1093/nar/11.21.7631 PMC326508

[33] Loo T. W., and Clarke D. M. (1995) P-glycoprotein: associations between domains and between domains and molecular chaperones. J. Biol. Chem. 270, 21839–21844 7545169 10.1074/jbc.270.37.21839

[34] Loo T. W., and Clarke D. M. (2015) The transmission interfaces contribute asymmetrically to the assembly and activity of human P-glycoprotein. J. Biol. Chem. 290, 16954–16963 25987565 10.1074/jbc.M115.652602 PMC4505440

[35] Loo T. W., and Clarke D. M. (1995) Membrane topology of a cysteine-less mutant of human P-glycoprotein. J. Biol. Chem. 270, 843–848 7822320 10.1074/jbc.270.2.843

[36] Loo T. W., Bartlett M. C., and Clarke D. M. (2004) Disulfide cross-linking analysis shows that transmembrane segments 5 and 8 of human P-glycoprotein are close together on the cytoplasmic side of the membrane. J. Biol. Chem. 279, 7692–7697 14670948 10.1074/jbc.M311825200

[37] Loo T. W., and Clarke D. M. (2014) Locking intracellular helices 2 and 3 together inactivates human P-glycoprotein. J. Biol. Chem. 289, 229–236 24275649 10.1074/jbc.M113.527804 PMC3879546

[38] Loo T. W., Bartlett M. C., and Clarke D. M. (2013) Human P-glycoprotein contains a greasy ball-and-socket joint at the second transmission interface. J. Biol. Chem. 288, 20326–20333 23733192 10.1074/jbc.M113.484550 PMC3711299

[39] Loo T. W., Bartlett M. C., and Clarke D. M. (2002) The “LSGGQ” motif in each nucleotide-binding domain of human P-glycoprotein is adjacent to the opposing walker A sequence. J. Biol. Chem. 277, 41303–41306 12226074 10.1074/jbc.C200484200

[40] Loo T. W., Bartlett M. C., and Clarke D. M. (2003) Drug binding in human P-glycoprotein causes conformational changes in both nucleotide-binding domains. J. Biol. Chem. 278, 1575–1578 12421806 10.1074/jbc.M211307200

[41] Loo T. W., and Clarke D. M. (1997) Drug-stimulated ATPase activity of human P-glycoprotein requires movement between transmembrane segments 6 and 12. J. Biol. Chem. 272, 20986–20989 9261097 10.1074/jbc.272.34.20986

[42] DeLano W. L. (2002) The PyMOL Molecular Graphics System, Schrödinger, LLC, New York

[43] Loo T. W., Bartlett M. C., and Clarke D. M. (2008) Arginines in the first transmembrane segment promote maturation of a P-glycoprotein processing mutant by hydrogen bond interactions with tyrosines in transmembrane segment 11. J. Biol. Chem. 283, 24860–24870 18596043 10.1074/jbc.M803351200 PMC3259837

[44] Loo T. W., Bartlett M. C., and Clarke D. M. (2009) Identification of residues in the drug-translocation pathway of the human multidrug resistance P-glycoprotein by arginine mutagenesis. J. Biol. Chem. 284, 24074–24087 19581304 10.1074/jbc.M109.023267 PMC2782001

[45] Loo T. W., Bartlett M. C., and Clarke D. M. (2004) The drug-binding pocket of the human multidrug resistance P-glycoprotein is accessible to the aqueous medium. Biochemistry 43, 12081–12089 15379547 10.1021/bi049045t

[46] Dorairaj S., and Allen T. W. (2007) On the thermodynamic stability of a charged arginine side chain in a transmembrane helix. Proc. Natl. Acad. Sci. U.S.A. 104, 4943–4948 17360368 10.1073/pnas.0610470104 PMC1829244

[47] Sarkadi B., Price E. M., Boucher R. C., Germann U. A., and Scarborough G. A. (1992) Expression of the human multidrug resistance cDNA in insect cells generates a high activity drug-stimulated membrane ATPase. J. Biol. Chem. 267, 4854–4858 1347044

[48] Urbatsch I. L., and Senior A. E. (1995) Effects of lipids on ATPase activity of purified Chinese hamster P-glycoprotein. Arch. Biochem. Biophys 316, 135–140 7840607 10.1006/abbi.1995.1020

[49] Loo T. W., and Clarke D. M. (1999) Identification of residues in the drug-binding domain of human P-glycoprotein: analysis of transmembrane segment 11 by cysteine-scanning mutagenesis and inhibition by dibromobimane. J. Biol. Chem. 274, 35388–35392 10585407 10.1074/jbc.274.50.35388

[50] Loo T. W., Bartlett M. C., Shi L., and Clarke D. M. (2012) Corrector-mediated rescue of misprocessed CFTR mutants can be reduced by the P-glycoprotein drug pump. Biochem. Pharmacol. 83, 345–354 22138447 10.1016/j.bcp.2011.11.014

[51] Van Goor F., Hadida S., Grootenhuis P. D., Burton B., Stack J. H., Straley K. S., Decker C. J., Miller M., McCartney J., Olson E. R., Wine J. J., Frizzell R. A., Ashlock M., and Negulescu P. A. (2011) Correction of the F508del-CFTR protein processing defect in vitro by the investigational drug VX-809. Proc. Natl. Acad. Sci. U.S.A. 108, 18843–18848 21976485 10.1073/pnas.1105787108 PMC3219147

[52] Chen Y. C. (2015) Beware of docking! Trends Pharmacol. Sci. 36, 78–95 25543280 10.1016/j.tips.2014.12.001

[53] Loo T. W., Bartlett M. C., and Clarke D. M. (2005) ATP hydrolysis promotes interactions between the extracellular ends of transmembrane segments 1 and 11 of human multidrug resistance P-glycoprotein. Biochemistry 44, 10250–10258 16042402 10.1021/bi050705j

[54] Loo T. W., Bartlett M. C., and Clarke D. M. (2003) Substrate-induced conformational changes in the transmembrane segments of human P-glycoprotein: direct evidence for the substrate-induced fit mechanism for drug binding. J. Biol. Chem. 278, 13603–13606 12609990 10.1074/jbc.C300073200

[55] Loo T. W., Bartlett M. C., and Clarke D. M. (2003) Methanethiosulfonate derivatives of rhodamine and verapamil activate human P-glycoprotein at different sites. J. Biol. Chem. 278, 50136–50141 14522974 10.1074/jbc.M310448200

[56] Loo T. W., and Clarke D. M. (2001) Cross-linking of human multidrug resistance P-glycoprotein by the substrate, tris-(2-maleimidoethyl)amine, is altered by ATP hydrolysis: evidence for rotation of a transmembrane helix. J. Biol. Chem. 276, 31800–31805 11429407 10.1074/jbc.M103498200

[57] Dey S., Ramachandra M., Pastan I., Gottesman M. M., and Ambudkar S. V. (1997) Evidence for two nonidentical drug-interaction sites in the human P-glycoprotein. Proc. Natl. Acad. Sci. U.S.A. 94, 10594–10599 9380680 10.1073/pnas.94.20.10594 PMC23414

[58] Wen P. C., Verhalen B., Wilkens S., Mchaourab H. S., and Tajkhorshid E. (2013) On the origin of large flexibility of P-glycoprotein in the inward-facing state. J. Biol. Chem. 288, 19211–19220 23658020 10.1074/jbc.M113.450114 PMC3696692

[59] Martin C., Berridge G., Mistry P., Higgins C., Charlton P., and Callaghan R. (1999) The molecular interaction of the high affinity reversal agent XR9576 with P-glycoprotein. Br. J. Pharmacol. 128, 403–411 10510451 10.1038/sj.bjp.0702807 PMC1571648

[60] Loo T. W., Bartlett M. C., and Clarke D. M. (2003) Permanent activation of the human P-glycoprotein by covalent modification of a residue in the drug-binding site. J. Biol. Chem. 278, 20449–20452 12711602 10.1074/jbc.C300154200

[61] Loo T. W., Bartlett M. C., and Clarke D. M. (2006) Transmembrane segment 1 of human P-glycoprotein contributes to the drug-binding pocket. Biochem. J. 396, 537–545 16492138 10.1042/BJ20060012 PMC1482826

[62] Loo T. W., and Clarke D. M. (2001) Defining the drug-binding site in the human multidrug resistance P-glycoprotein using a methanethiosulfonate analog of verapamil, MTS-verapamil. J. Biol. Chem. 276, 14972–14979 11279063 10.1074/jbc.M100407200

[63] Bhatia A., Schäfer H. J., and Hrycyna C. A. (2005) Oligomerization of the human ABC transporter ABCG2: evaluation of the native protein and chimeric dimers. Biochemistry 44, 10893–10904 16086592 10.1021/bi0503807

[64] Jin M. S., Oldham M. L., Zhang Q., and Chen J. (2012) Crystal structure of the multidrug transporter P-glycoprotein from *Caenorhabditis elegans*. Nature 490, 566–569 23000902 10.1038/nature11448 PMC3482266

[65] Loo T. W., and Clarke D. M. (1999) The human multidrug resistance P-glycoprotein is inactive when its maturation is inhibited: potential for a role in cancer chemotherapy. FASEB J. 13, 1724–1732 10506575 10.1096/fasebj.13.13.1724

[66] Chen E. Y., Bartlett M. C., and Clarke D. M. (2000) Cystic fibrosis transmembrane conductance regulator has an altered structure when its maturation is inhibited. Biochemistry 39, 3797–3803 10736180 10.1021/bi992620m

[67] Chen E. Y., Bartlett M. C., Loo T. W., and Clarke D. M. (2004) The ΔF508 mutation disrupts packing of the transmembrane segments of the cystic fibrosis transmembrane conductance regulator. J. Biol. Chem. 279, 39620–39627 15272010 10.1074/jbc.M407887200

[68] Wang Y., Loo T. W., Bartlett M. C., and Clarke D. M. (2007) Correctors Promote Maturation of Cystic Fibrosis Transmembrane Conductance Regulator (CFTR)-processing Mutants by Binding to the Protein. J. Biol. Chem. 282, 33247–33251 17911111 10.1074/jbc.C700175200

[69] Loo T. W., Bartlett M. C., and Clarke D. M. (2008) Correctors promote folding of the CFTR in the endoplasmic reticulum. Biochem. J. 413, 29–36 18361776 10.1042/BJ20071690

